# Extraction Optimization and Characterization of Cellulose Nanocrystals from Apricot Pomace

**DOI:** 10.3390/foods12040746

**Published:** 2023-02-08

**Authors:** Ekin Dinçel Kasapoğlu, Sibel Kahraman, Fatih Tornuk

**Affiliations:** 1 Food Technology Program, Anadolu Bil Vocational School of Higher Education, İstanbul Aydın University, 34295 İstanbul, Turkey; 2 Gastronomy and Culinary Arts Department, Fine Arts Faculty, İstanbul Aydın University, 34295 İstanbul, Turkey; 3 Food Engineering Department, Chemical and Metallurgical Engineering Faculty, Yıldız Technical University, 34220 İstanbul, Turkey

**Keywords:** apricot pomace, cellulose nanocrystals, agro waste, optimization

## Abstract

Apricot pomace (AP) is lignocellulosic agro-industrial waste that could be considered a good source for cellulose-based, value-added compounds. In this study, conditions for cellulose nanocrystals’ (CNCs) extraction from apricot pomace (AP) were optimized using Response Surface Methodology (RSM) based on the extraction yield, and the resulting CNC was characterized using Fourier transform infrared (FTIR) spectroscopy, Scanning Electron Microscopy (SEM), Transmittance Electron Microscopy (TEM), Thermogravimetric Analysis (TGA), and X-Ray Diffraction (XRD). The maximum CNC yield (34.56%) was obtained at a sulfuric acid concentration of 9.5 M within 60 min. FTIR analysis showed that noncellulosic components were gradually removed from the pomace. A morphological analysis of the nanocrystal was performed using SEM and TEM. CNCs were in the range of 5–100 µm in diameter and appeared as individual fibers. TGA analysis of the CNC sample revealed good thermal stability around 320°C. The crystalline index (%CI) of the CNC obtained from AP was determined to be 67.2%. In conclusion, this study demonstrated that AP could be considered a sustainable source for value-added compounds such as CNCs to contribute to a circular economy.

## 1. Introduction

As is known, a large amount of waste and byproducts are generated by the food and agricultural activities [1]. The need for an effective use of raw materials and a reduction in waste amounts has become an important issue for researchers in recent years [2]. Wastes from fruit processing technology constitute a significant portion of food processing residues. Fruit pomace is the major residue remaining from fruit juice production [3].

Every year, apricot processing plants produce more than 300.000 tons of byproducts, namely, apricot skin, apricot pomace (AP), stones, shells, kernels, and kernel skin. The part called AP consists of fruit flesh and fruit peel [4]. AP contains carbohydrates, proteins, vitamins, minerals, water, as well as all water-soluble components together with all water-insoluble compounds [5,6,7].

The production of environmentally compatible and value-added materials from food and agricultural industry wastes has attracted great attention in recent years. The terms such as “cellulose nanowhiskers”, “cellulose nanocrystals (CNCs)”, “nanocrystalline cellulose (NCC)”, “monocrystals”, “microcrystals”, and “microcrystallites” are commonly used to describe nano-scale cellulose derivatives [8]. Many studies on the use of polysaccharides such as cellulose, obtained from lignocellulosic resources, such as reinforcing agents, especially in natural polymer matrices, have gained great importance recently [9].

CNCs are needle-shaped nanometric particles extracted from cellulose and have a crystalline structure less than or equal to 100 nm in size [10]. The biocompatibility, biodegradability, availability, and low cost of CNCs obtained from renewable natural sources have led to their use in a variety of applications [11]. The acid hydrolysis method is the most effective and common method for obtaining CNCs [12], and it is often used as a reinforcement agent in barrier and film applications [13]. Additionally, CNCs have been used as templates for various materials, as separation membranes, and as active materials in batteries and supercapacitors. They have also been used in the production of electroactive polymers for use in implantable medical devices, pharmaceuticals, fibers, and textile electronic components [14].

Although a variety of natural fibers (such as coconut husk fibers [15], cassava bagasse [16], banana rachis [17], mulberry bark [18], soybean pods [19], soy hulls [20], and cornstalks [21]) have been investigated in detail, the use of AP as a natural source used to produce CNCs has not yet been widely explored. Therefore, in this study, CNC production conditions for AP were optimized using Response Surface Methodology (RSM) to maximize the extraction yield and the CNCs obtained from the optimized conditions. The conditions were then characterized using Fourier transform infrared (FTIR), Scanning Electron Microscopy (SEM), Transmittance Electron Microscopy (TEM), Thermogravimetric Analysis (TGA), and X-Ray Diffraction (XRD).

## 2. Materials and Methods

### 2.1. Materials

Fresh apricot (*Prunus armeniaca*) pomace (AP) was provided from a fruit concentrate producer (Anadolu Etap Penkon Corporation, Mersin, Turkey). The material was transferred to the laboratory in plastic bags immediately and dried in an oven (Nuve Incubator EN 120, Ankara, Turkey) at 105 °C until a 5.6% moisture level was reached. Then, the dried pomace was powdered and stored at −20 °C in polyethylene bags prior to the experiments.

All chemicals (sodium hydroxide, sodium hypochlorite, sulphuric acid, glacial acetic acid, cellulose membrane, and cellulose filter paper) were supplied by Sigma–Aldrich Corporation (St. Louis, MO, USA).

### 2.2. Purification of Cellulose and Cellulose Nanocrystals (CNCs) from AP

**Figure 1:**
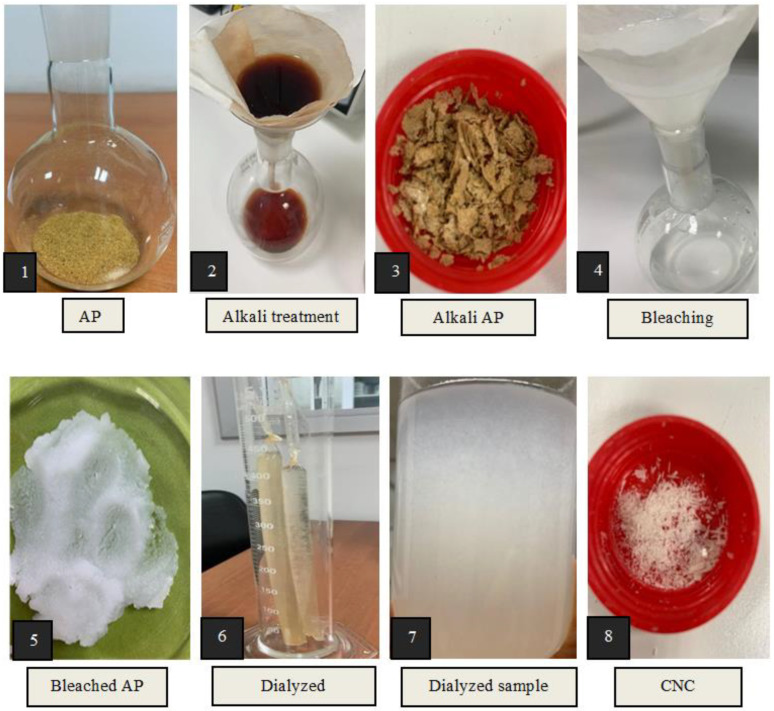
The steps for the extraction of cellulose and CNC from AP.

Cellulose was purified by removing pectic polysaccharides, hemicellulose, and lignin from the raw material via sequential alkaline treatment, bleaching, and acid hydrolysis processes, as suggested by [22,23,24]. Then, the CNCs were prepared from the purified cellulose using acid hydrolysis with minor modifications [24]. The steps for extracting cellulose and CNC from AP are depicted in Figure 1.

#### 2.2.1. Alkaline Process

The alkaline process was applied for the extraction of cellulose by removing lignin and hemicellulose from AP using the methods described by Johar et al. [22]. The AP powder (1:20 *w*:*v*) was treated with a 4% (*w*/*v*) NaOH solution for 2 h at 120 °C under mechanical stirring. Then, the solid was filtered using rough filter paper and washed several times with distilled water to remove the alkaline residues. The sample was then dried at 37 °C for 24 h in an air-circulating oven, as suggested by [22].

#### 2.2.2. Bleaching 

The alkali-treated sample was then exposed to the bleaching process. For this purpose, the sample was incorporated with 2% NaOCI solution (prepared in acetate buffer) at a ratio of 1:10 (*w*:*v*) and stirred for 3 h at 120 °C. After this period, distilled water was added to the sample for cooling, and the suspension was filtered. The bleaching process was repeated thrice.

#### 2.2.3. Experimental Design for Acid Hydrolysis

In this study, to maximize the cellulose purification yield from the AP, a central composite-based experimental design using a Response Surface Methodology was employed. For this purpose, acid concentration (X1) and extraction time (X2) were selected as independent variables, and CNC extraction yield (%) was chosen as the response. Table 1 shows the codified values and independent variables used for optimization. Consequently, a total of 12 experiments were performed with 4 factorial points, 4 axial points, and 4 replicates of the central points, as shown in Table 2. This step was repeated three times for each experimental point. At the end of the acid hydrolysis process, 100 mL of cold distilled water was added to the reaction mixture to stop the reaction. Then, centrifugation at 4000× *g* rpm was performed for 10 min to remove the acidic medium from the hydrolyzed material. Then, the precipitate was membrane dialyzed for 48 h to remove the residual acids and obtain the material at a neutral pH. After this treatment, CNC solutions were freeze-dried and then stored at 4 °C for characterization.

#### 2.2.4. Determination of CNC Extraction Yield 

The extraction yield was calculated by dividing the final weight of the dried AP by the initial weight of the AP. The results were given as percent as dry matter base.

### 2.3. Characterization of CNC 

The CNC sample obtained from AP at the optimized reaction conditions was characterized using FTIR, TEM, SEM, TGA, and XRD.

#### 2.3.1. FTIR 

Fourier transform infrared spectra of the CNC, AP, alkali AP, and bleached AP were obtained using a FT-IR spectrophotometer (FT-IR, Cary 630, Agilent, Danbury, CT, USA). Firstly, the samples were transferred onto KBr discs. FTIR spectral analysis was recorded on an Agilent FTIR spectrophotometer in the wavelength range of 400–4000 cm^−1^. Commercial cellulose filter paper (Sigma–Aldrich, St. Louis, MO, USA) was also analyzed to determine its similarity with the resulting CNC. 

#### 2.3.2. TEM

Using a JEOL JEM-1220 TE TEM tool running at 80 kV (JEOL Ltd., Tokyo, Japan), the dimensions of the AP-based CNC were evaluated. For this, a carbon film was placed over a drop of CNC suspension (0.001% by weight) that had been collected on the surface of a copper grid. For the TEM contrast, cellulose nanocrystals (CNCs) were stained negatively in a 2% wt uranyl acetate solution for 10 s and allowed to air dry at room temperature.

#### 2.3.3. SEM 

SEM analysis of the CNC obtained from AP was performed using the JEOL SEM (JSM-7001F, Japan) to determine the surface morphology. Before analysis, the sample was covered in a layer of gold. The accelerating voltage was 15 kV.

#### 2.3.4. TGA

Thermal degradation characteristics of dried AP, alkali AP, bleached AP, and CNC were determined using a Mettler Toledo model TGA/SDTA851e device under a nitrogen atmosphere. The flow rate, temperature range, and heating rate were set at 30 mL min^−1^, 30 to 600 °C, and 10 °C min^−1^, respectively.

#### 2.3.5. XRD

The crystal structure of the extracted CNC was evaluated through XRD analysis. The crystallinity of CNCs obtained from apricot pomace was determined using an X-ray diffractometer (Bruker D8 Discover, Billerica, MA, USA) set to 40 kV, 30 mA, and CuKa radiation (1.5406). X-ray diffractograms of CNCs were collected at a 2*θ* range of 10° to 60°. According to Segal’s method [25], the CNCs’ crystallinity index (*CI*%) is determined using the following formula:

(1) $$CI\;\left(\%\right)=\frac{I_{002}-I_{am}}{I_{002}}\times 100$$

where *I*_002_ is the peak density of the crystalline material at 2*θ* ≈ 22.5 and *I_am_* is the peak density of the amorphous material at 2*θ* ≈ 18.

## 3. Results and Discussion

### 3.1. Yield of Cellulose Nanocrystals from Apricot Pomace

Acidic extraction was applied for 30 to 60 min, and acid concentrations were between 9 M and 10 M. The temperature was selected as 45 °C. The predicted response Y for the CNC extraction yield was shown by the second-order polynomial equation. It was given in terms of coded values, as in the following equation.

Yield of cellulose nanocrystals (Y) = + 33.1 + 2.13X + 1.54Y − 0.3400 XY − 9.59X^2^ + 0.9838 Y^2^ (2)

**Figure 2:**
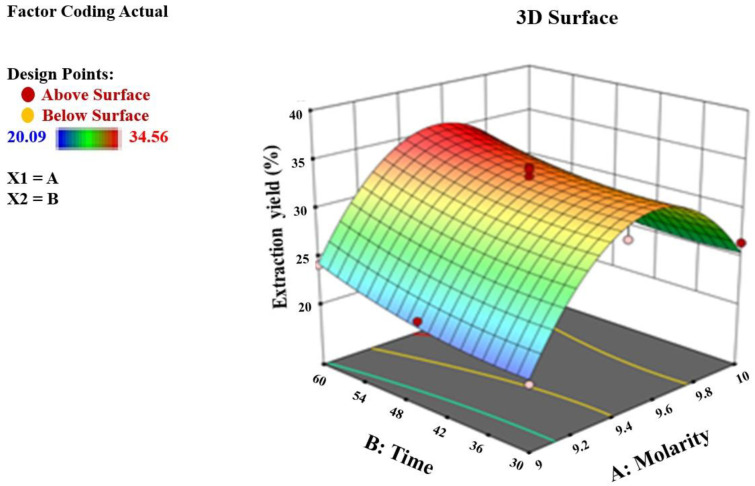
The interaction effect of the extraction variables on the yield of CNCs.

Table 2 lists the apricot pomace extract extraction yield (%) on RSM experimental points. The samples’ yields varied from 20.09% to 34.56%, with the greatest result being a 9.5 M acid concentration and 60 min of treatment. The relationship between extraction factors and CNC yield is seen in Figure 2. The graph illustrates how the yield of the CNCs’ values of the extracts increased with time.

As shown in Table 3, there was a quadratic relationship between the extraction yield of CNCs and the extraction parameters (acid concentration and time), with a good regression coefficient (R^2^ = 0.9590). The model is valid since the F value for the lack of fit was insignificant (*p* > 0.05). The adjusted determination coefficient value (adjusted R^2^ = 0.9248) further demonstrated the model’s high level of significance. The coefficient of variation (CV), which had a very low value of 5.08, also made it evident that the experimental data had a high degree of precision and a lot of dependability. Moreover, it had an extremely low model *p* value (Prob > F). The chosen quadratic model for the CNC yield is shown in Table 3 along with the analysis of variance (ANOVA), and the estimated regression coefficients and their significance test for the second-order model are shown in Table 4. 

It is also important to note that the type of cellulose source used can also affect the outcome of the suspension. For example, using a pure cellulose source such as wood pulp produces a higher yield of CNCs compared to using a mixed cellulose source such as plant fibers. Furthermore, the size and shape of the cellulose whiskers can also be manipulated by adjusting the acid concentration and hydrolysis duration. For example, a higher acid concentration and longer hydrolysis duration results in smaller, more uniform sized whiskers. The mechanical properties of the final cellulose nanocrystal suspension can also be affected by these variables, with a higher acid concentration and longer hydrolysis duration resulting in stronger and more flexible materials. Overall, the precise control of the sulfuric acid concentration and hydrolysis duration is crucial for the successful production of CNCs [26].

According to a recent study, it was determined that the most important parameters in CNC yield were the concentration of the acid and the time of the analysis. In a study, the CNC yield of passion fruit peels was found to be 52% in conditions where the sulfuric acid concentration was 8.4 M and the hydrolysis time was 60 min [27]. There have been reports of varying CNC yields from various raw materials in the literature. For example, Jiang et al. [28] found that CNC yield from a tomato peel was 15.7%. Additionally, the highest yield of CNC extraction from an onion peel studied using a 45% (8 M) acid concentration for 45 min was reported as 48.6% [29].

Gao et al. [30] and Marett et al. [31] reported that CNC yields were 29.2 % and 50%, respectively, from *Calotropis gigantea* fiber and pistachio shell, and the yield was dependent on raw material, acid concentration, and extraction time. In the study conducted by Dos Santos et al. [32], CNCs were produced from pineapple leaves via acid hydrolysis, using 20 mL of H_2_SO_4_ (9.17 M) for 5, 30, or 60 min at 45 °C. The crystallinity index, FTIR, morphology (shape and size), and thermal stability of the obtained CNCs were characterized. Among the hydrolysis conditions carried out, the optimum extraction time was determined as 30 min. This study highlighted the potential of using pineapple leaves as a raw material for CNC production, as well as the importance of optimizing the hydrolysis conditions for achieving the maximum yield and quality of CNCs. In another study, Wijaya et al. [33] stated that the CNC yield reached the highest value (77.48%) at a sulfuric acid concentration of 56.21% and a temperature of 43°C for the production of CNC. This study also emphasized the importance of acid concentration and temperature in determining the yield of CNCs, with a sulfuric acid concentration of 56.21% and a temperature of 43°C resulting in the highest yield of 77.48%. Furthermore, 11.21 M sulfuric acid concentration and 10 min of extraction time were applied to obtain CNCs from mango seed, and the CNC yield was determined as 22.8% [34]. 

### 3.2. FTIR 

**Figure 3:**
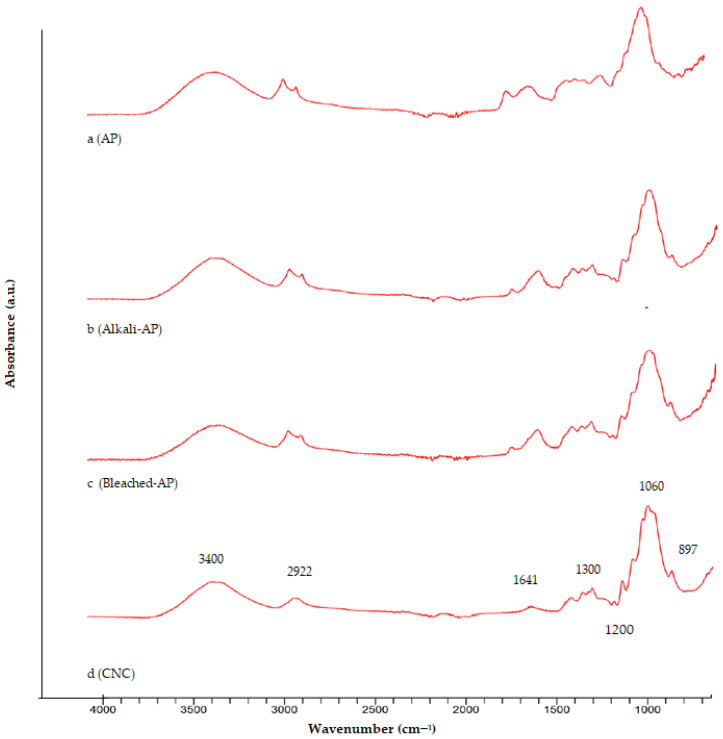
FTIR spectra of (**a**) AP, (**b**) alkali AP, (**c**) bleached AP, (**d**) CNC.

FTIR spectra of the CNC, AP, alkali AP, and bleached AP are shown in Figure 3. The peak around 3300–3400 cm^−1^ was seen in all samples and correlates with the C-H and O-H groups. When compared to other samples, the peak of the O-H stretching vibration in the CNC has a higher transmittance. The breaking of some glycosidic bonds and the loss of some amorphous areas in the cellulose structure are the main causes of the increase in hydrogen bonds. The peak at 897 cm^−1^ is caused by the glycosidic link between the monomers of the cellulose. The peak around 1640 cm^−1^ corresponding to the water absorption was observed in all samples, and the peak around 1300 cm^−1^ expresses the structural arrangement of the C-H group [35]. The absorption bending in the C-H, O-H, and CH_2_ groups occurs at peaks in the range of 1266–1200 cm^−1^. A peak around 1700 cm^−1^ indicates the presence of acetyl and ester groups in hemicellulose or carboxylic acid groups in the ferulic and *p*-coumaric components of lignin. [20]. The peak around 1700 cm^−1^ in the spectra of untreated AP cannot be seen for CNC because of the elimination of noncellulosic parts. It is observed that, immediately after acid hydrolysis, hemicellulose peaks in cellulose nanocrystals in the FTIR spectra completely disappear with the breakdown of hemicelluloses into pentose sugars. 

**Figure 4:**
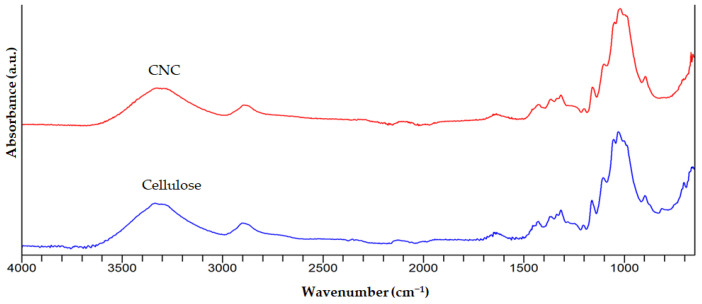
FTIR spectra of CNC and cellulose.

As can be seen from the spectra in Figure 3, hemicellulose and lignin were removed from the AP by using treatment with HCl, NaOH, and NaOCl to obtain cellulose. These results were in accordance with the results reported in previous studies [22,34,36,37]. In Figure 4, the similarity of the FTIR spectrum of CNC and cellulose was 98.01%, showing the success of the extraction.

### 3.3. SEM

**Figure 5:**
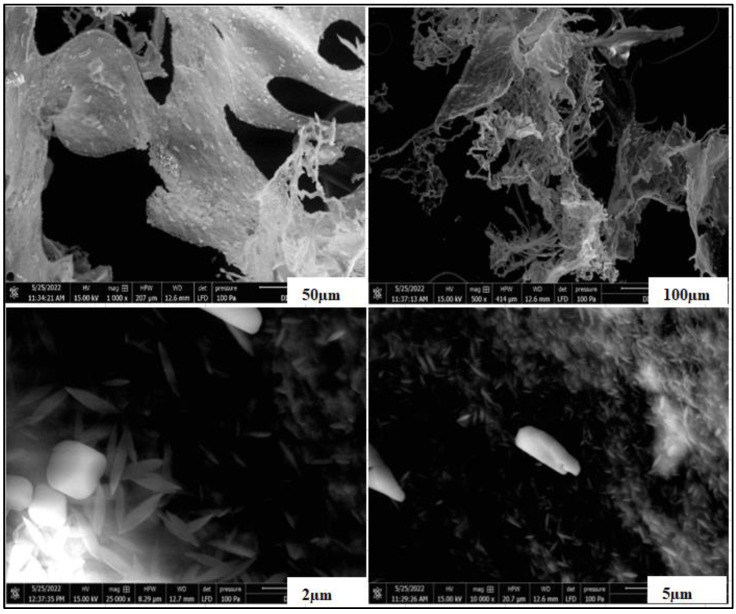
SEM images of CNC from AP.

SEM analysis was performed to determine whether large aggregate structures were formed that could affect the mechanical and water barrier properties of the CNC. After the acid hydrolysis process, CNCs were in the range of 5–100 µm in diameter and appeared as individual fibers. The changes which occurred upon performing the acid hydrolysis step are shown in Figure 5. After the acidic treatment, the raw cellulose fiber structure was damaged, and the smooth surface became rougher. The most obvious reason for clumping between CNC particles was the presence of strong intermolecular hydrogen bonds. The SEM findings were in accordance with the results of Cheng et al. [38], who reported that CNCs obtained from the acid hydrolysis had an irregular shape and size. The hydrolytic breakage of the glycosidic bond during acidification, which results in the breakdown of amorphous areas, was also linked to the tiny CNC particle size [39].

### 3.4. TEM

**Figure 6:**
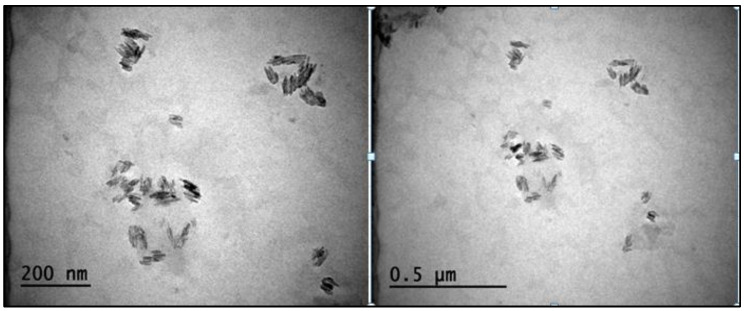
TEM images of CNC from apricot pomace at 200 nm and 0.5 µm size.

TEM micrographs of the CNCs are shown in Figure 6. The CNCs contained structures that resembled rods, as shown in the TEM images. Figure 6 also showed that the CNC was slightly clustered, with some particles clumping together. It is generally known that the fiber supply and acid hydrolysis conditions affect the shape of CNCs [40]. Similar findings for CNCs obtained from oil palm trunk biomass have also been reported. The appearance of laterally clustered crystals noticed in TEM images was associated with strong hydrogen bonding between the fibers. These aggregate structures could be found in suspension or even come together during sample preparation for TEM [41]. It has been demonstrated that dried CNCs were more agglomerated than non-dried ones [42]. Additionally, in the freeze-drying process, the production of ice crystals was primarily responsible for CNC aggregation [43]. Furthermore, the acid hydrolysis conditions used in the production of CNCs can also affect the shape and aggregation of the CNCs. CNCs produced under harsher acid hydrolysis conditions tended to form more rod-like shaped CNCs which were more aggregated compared to those produced under milder conditions.

### 3.5. TGA 

**Figure 7:**
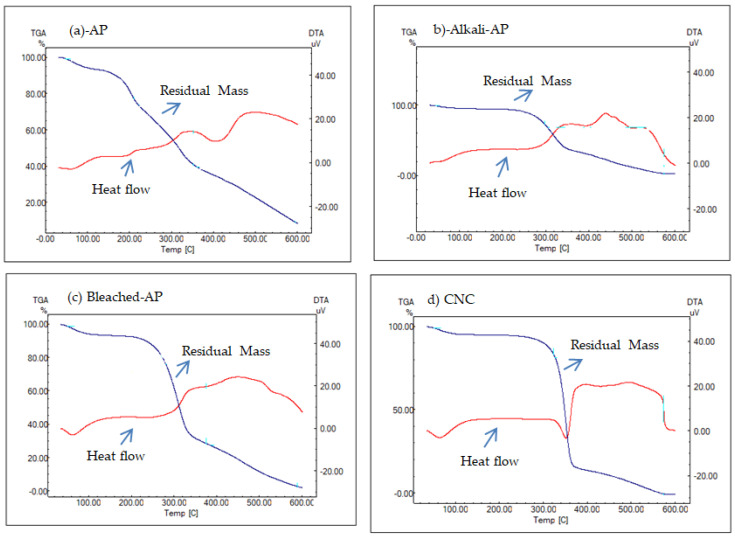
TGA curves for AP (apricot pomace), alkali AP, bleached AP, and CNC.

TGA was used to evaluate the thermal stability of the CNCs obtained from AP. TGA graphics of AP, alkali-treated AP, and bleached AP were also obtained for comparison. The results showed that the TGA and DTA profiles were correlated with the weight loss of the samples upon continuous heating to 600 °C. The elimination of hemicellulose, lignin, and pectin using alkaline and bleaching procedures might be the major reason behind the higher thermal degradation beginning temperature in the CNC [20,36,44]. Additionally, it was found that the thermal stability of the CNC was affected by the presence of impurities such as hemicellulose, lignin, and pectin. These impurities likely lowered the thermal degradation beginning temperature in the CNC. The alkali and bleaching treatments were found to effectively remove these impurities, resulting in a higher thermal degradation beginning temperature in the CNC. This could be seen in the TGA and DTA profiles, which showed a correlation between the weight loss of the samples and the temperature at which they were heated. Overall, the TGA analysis provided a valuable insight into the thermal stability of the CNCs and the effect of impurities and treatments on this stability. It has been reported that sulfuric acid treatment could cause a decrease in the thermal stability of CNC. This may be due to the formation of sulfate groups on the surface of the cellulose, which created catalytic thermal degradation reactions [45]. The initial weight loss due to the removal of moisture from the cellulose fibers was determined to be 15% below 323 °C. The first change could be explained by the evaporation of water and other volatile compounds. Therefore, the initial weight loss could be related to a decrease in the moisture content of the samples. More significant weight loss was observed at higher temperatures. Major weight loss (80%) occurred when heating the sample (from 500 °C to 600 °C). Neutralizing the acid-sulfated groups of CNC with NaOH has been proven to increase the thermal stability of CNCs [46]. For unhydrolyzed fiber (Figure 7a), there was a rapid weight loss around 300 °C. The main peak after the hydrolysis of cellulose was observed at around 323 °C. The initial degradation temperatures were found to be around 210, 297, 271, and 323 °C for the AP, alkali AP, bleached AP, and CNC, respectively. For the AP, alkali AP, bleached AP, and CNC, the starting degradation temperatures were around 210, 297, 271, and 323 °C, respectively. Compared to AP, the CNC’s initial decomposition temperature (323 °C) was greater (Figure 7a–d). In comparison to cellulose, these components (hemicellulose, lignin, and pectin) had lower degradation temperatures, and it has been demonstrated that sequentially removing these structures improved the thermal structure and stability of the CNCs [47].

### 3.6. XRD

**Figure 8:**
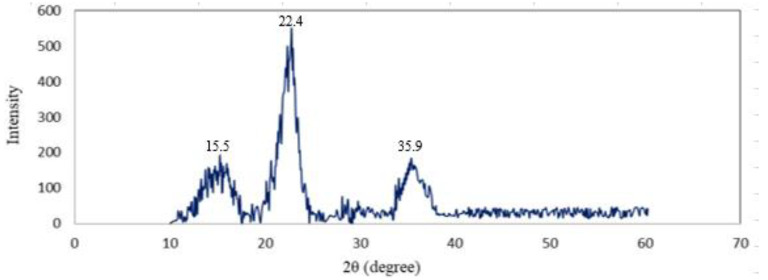
X-ray diffractogram of the CNC.

The CNC’s high crystallinity is the most important factor in determining its thermal stability and mechanical properties. The degree of crystallinity of the CNC is shown in Figure 8. According to the results of the XRD analysis, CNC peaks compatible with cellulose structure were recorded as 2*θ* = 15.5°, 22.4°, and 35.9°, respectively. The crystalline index (CI%) of the CNC obtained from AP was determined as 67.2%. It was shown that the extending of the acid hydrolysis period not only reduces the amorphous regions of the cellulose but also reduces the crystallinity and yield by destroying the crystalline regions of the cellulose [48]. By acting on the amorphous regions of the cellulose, H_3_O^+^ ions hydrolyzed the glycosidic bonds that caused an increase in crystallinity [18]. The crystallinity index value in our study was higher than pineapple leaf fibers’ (54%) crystallinity index value [49] but lower than the specified values for potato peel CNCs (85%) [36], sisal fibers (75%) [47], and cotton CNCs (91%) [50].

## 4. Conclusions

In this study, CNC was successfully purified from AP with a high yield through an optimization of the extraction conditions using RSM. Microscopic observations of the CNC, which was obtained with the highest yield, were carried out. The maximum yield (34.56%) of CNC was obtained using a sulfuric acid concentration of 9.5 M within 60 min. The CNC had a structure similar to the rod-like crystals. The crystalline index value was calculated as 67.1%. The sample exhibited high heat stability until 300 °C. In conclusion, this study revealed the potential of CNC obtained from AP as a valuable component which could be utilized in various fields such as food packaging, electronics, and nanosensors, as well as tissue engineering and enzyme immobilization.

## Figures and Tables

**Table 1:** Codified values and independent variables used for optimization.

Independent Variables	Units	Codes	Coded Levels		
			−1	0	1
Acid concentration	M	X1	9	9.5	10
Time	Min	X2	30	45	60

**Table 2:** Apricot pomace extract extraction yield (%) on RSM experimental points.

		Factor 1	Factor 2	Response 1
Std	Run	Acid Concentration (M)	Time (min)	Extraction Yield (%)
7	1	9.5	30	32.09
11	2	9.5	45	33.4
10	3	9.5	45	34.32
9	4	9.5	45	32.55
2	5	10	30	26.5
5	6	9	45	22.1
3	7	9	60	24.15
12	8	9.5	45	34.1
8	9	9.5	60	34.56
6	10	10	45	23.4
1	11	9	30	20.09
4	12	10	60	29.2

**Table 3:** ANOVA for the chosen quadratic model to optimize apricot pomace CNC extraction yield (%).

Source	CNC Yield (%)					
	SS	DF	MS	*F*-Value	*p*-Value	
**Model**	301.81	5	60.36	28.07	0.0004	Significant
**Residual**	12.9	6	2.15			
Lack of Fit	10.99	3	3.66	5.75	0.0923	Not significant
Pure Error	1.91	3	0.6369			
**Core Total**	314.71	11				

R^2^ = 0.9590; R^2^ adj = 0.9248; R^2^ pred = 0.6763; C.V.% = 5.08; adequate precision = 14.5952; DF degree of freedom, SS sum of squares, MS mean square. Data are means of triplicates. Means with different factors are significantly different at *p* < 0.05.

**Table 4:** Estimation of the regression coefficients and their significance analysis for the CNC quadratic model.

Source	Sum of Squares	df	Mean Square	*F*-Value	*p*-Value
X_1_-Acid concentration	27.14	1	27.14	12.62	0.012
X_2_-Time	14.2	1	14.2	6.6	0.0424
X_1_ X_2_	0.4624	1	0.4624	0.215	0.6592
X_1_^2^	245.31	1	245.31	114.07	<0.0001
X_2_^2^	2.58	1	2.58	1.2	0.3153

DF degree of freedom, SS sum of squares, MS mean square. Data are means of triplicates. Means with different factors are significantly different at *p* < 0.05.

## Data Availability

Data available on request.

## References

[1] Mak T.M., Xiong X., Tsang D.C., Iris K.M., Poon C.S. (2020). Sustainable food waste management towards circular bioeconomy: Policy review, limitations and opportunities. Bioresour. Technol..

[2] Akhone M.A., Bains A., Tosif M.M., Chawla P., Fogarasi M., Fogarasi S. (2022). Apricot Kernel: Bioactivity, Characterization, Applications, and Health Attributes. Foods.

[3] Banerjee J., Singh R., Vijayaraghavan R., MacFarlane D., Patti A.F., Arora A. (2017). Bioactives from fruit processing wastes: Green approaches to valuable chemicals. Food Chem..

[4] Kasapoglu E.D., Kahraman S., Tornuk F. (2021). Optimization of ultrasound assisted antioxidant extraction from apricot pomace using response surface methodology. Food Meas. Charact..

[5] Haciseferogullari H., Gezer I., Özcan M.M., MuratAsma B. (2007). Post-harvest chemical and physical–mechanical properties of some apricot varieties cultivated in Turkey. J. Food Eng..

[6] Akin E.B., Karabulut I., Topcu A. (2008). Some compositional properties of main Malatya apricot (Prunus armeniaca L.) varieties. Food Chem..

[7] Chauhan S.K., Tyagi S.M., Singh D. (2001). Pectinolytic liquefaction of apricot, plum, and mango pulps for juice extraction. Int. J. Food Prop..

[8] Siqueira G., Bras J., Dufresne A. (2010). Cellulosic bionanocomposites: A review of preparation, properties and applications. Polymers..

[9] Kalia S., Dufresne A., Cherian B.M., Kaith B.S., Avérous L., Njuguna J., Nassiopoulos E. (2011). Cellulose-based bio-and nanocomposites: A review. Int. J. Polym. Sci..

[10] de Oliveira Junior S.D., Asevedo E.A., de Araujo J.S., Brito P.B., dos Santos Cruz Costa C.L., de Macedo G.R., dos Santos E.S. (2020). Enzymatic extract of Aspergillus fumigatus CCT 7873 for hydrolysis of sugarcane bagasse and generation of cellulose nanocrystals (CNC). Biomass Convers. Biorefin..

[11] Malainine M.E., Dufresne A., Dupeyre D., Mahrouz M., Vuong R., Vignon M.R. (2003). Structure and morphology of cladodes and spines of *Opuntia ficus-indica*. Cellulose extraction and characterisation. Carbohydr. Polym..

[12] Peng B.L., Dhar N., Liu H.L., Tam K.C. (2011). Chemistry and applications of nanocrystalline cellulose and its derivatives: A nanotechnology perspective. Can. J. Chem. Eng..

[13] Mariano M., El Kissi N., Dufresne A. (2014). Cellulose nanocrystals and related nanocomposites: Review of some properties and challenges. J. Polym. Sci. B. Polym. Phys..

[14] Silvério H.A., Neto W.P.F., Dantas N.O., Pasquini D. (2013). Extraction and characterization of cellulose nanocrystals from corncob for application as reinforcing agent in nanocomposites. Ind. Crops Prod..

[15] Morais J.P.S., de Freitas Rosa M., Nascimento L.D., do Nascimento D.M., Cassales A.R. (2013). Extraction and characterization of nanocellulose structures from raw cotton linter. Carbohydr. Polym..

[16] Pasquini D., de Morais Teixeira E., da Silva Curvelo A.A., Belgacem M.N., Dufresne A. (2010). Extraction of cellulose whiskers from cassava bagasse and their applications as reinforcing agent in natural rubber. Ind. Crops Prod..

[17] Zuluaga R., Putaux J.L., Cruz J., Vélez J., Mondragon I., Gañán P. (2009). Cellulose microfibrils from banana rachis: Effect of alkaline treatments on structural and morphological features. Carbohydr. Polym..

[18] Li R., Fei J., Cai Y., Li Y., Feng J., Yao J. (2009). Cellulose whiskers extracted from mulberry: A novel biomass production. Carbohydr. Polym..

[19] Wang B., Sain M. (2007). Isolation of nanofibers from soybean source and their reinforcing capability on synthetic polymers. Compos. Sci. Technol..

[20] Alemdar A., Sain M. (2008). Biocomposites from wheat straw nanofibers: Morphology, thermal and mechanical properties. Compos. Sci. Technol..

[21] Reddy N., Yang Y. (2005). Structure and properties of high quality natural cellulose fibers from cornstalks. Polymer.

[22] Johar N., Ahmad I., Dufresne A. (2012). Extraction, preparation and characterization of cellulose fibres and nanocrystals from rice husk. Ind. Crops Prod..

[23] Szymańska-Chargot M., Chylińska M., Gdula K., Kozioł A., Zdunek A. (2017). Isolation and characterization of cellulose from different fruit and vegetable pomaces. Polymer.

[24] Sheltami R., Ahmad I. (2021). Acid Hydrolysis Process of Mengkuang Cellulose. Malays. J. Anal. Sci..

[25] Segal L., Creely J.J., Martin A.E., Conrad C.M. (1959). An empirical method for estimating the degree of crystallinity of native cellulose using X-ray diffractometer. Text. Res. J..

[26] Bondeson D., Mathew A., Oksman K. (2006). Optimization of the isolation of nanocrystals from microcrystalline cellulose by acid hydrolysis. Cellulose.

[27] Wijaya C.J., Saputra S.N., Soetaredjo F.E., Putro J.N., Lin C.X., Kurniawan A., Ismadji S. (2017). Cellulose nanocrystals from passion fruit peels waste as antibiotic drug carrier. Carbohydr. Polym..

[28] Jiang F., Hsieh Y.L. (2015). Cellulose nanocrystal isolation from tomato peels and assembled nanofibers. Carbohydr. Polym..

[29] Rhim J.W., Reddy J.P., Luo X. (2015). Isolation of cellulose nanocrystals from onion skin and their utilization for the preparation of agar-based bio-nanocomposites films. Cellulose.

[30] Gao A., Chen H., Tang J., Xie K., Hou A. (2020). Efficient extraction of cellulose nanocrystals from waste *Calotropis gigantea* fiber by SO_4_^2-^/TiO_2_ nano-solid superacid catalyst combined with ball milling exfoliation. Ind. Crops Prod..

[31] Marett J., Aning A., Foster E.J. (2017). The isolation of cellulose nanocrystals from pistachio shells via acid hydrolysis. Ind. Crops Prod..

[32] Dos Santos R.M., Neto W.P.F., Silvério H.A., Martins D.F., Dantas N.O., Pasquini D. (2013). Cellulose nanocrystals from pineapple leaf, a new approach for the reuse of this agro-waste. Ind. Crops Prod..

[33] Wijaya C.J., Ismadji S., Aparamarta H.W., Gunawan S. (2019). Optimization of cellulose nanocrystals from bamboo shoots using Response Surface Methodology. Heliyon.

[34] Henrique M.A., Silvério H.A., Neto W.P.F., Pasquini D. (2013). Valorization of an agro-industrial waste, mango seed, by the extraction and characterization of its cellulose nanocrystals. J. Environ. Manag..

[35] Sun X.F., Xu F., Sun R.C., Fowler P., Baird M.S. (2005). Characteristics of degraded cellulose obtained from steam-exploded wheat straw. Carbohydr. Res..

[36] Chen D., Lawton D., Thompson M.R., Liu Q. (2012). Biocomposites reinforced with cellulose nanocrystals derived from potato peel waste. Carbohydr. Polym..

[37] Sheltami R.M., Abdullah I., Ahmad I., Dufresne A., Kargarzadeh H. (2012). Extraction of cellulose nanocrystals from mengkuang leaves (*Pandanus tectorius*). Carbohydr. Polym..

[38] Cheng M., Qin Z., Chen Y., Hu S., Ren Z., Zhu M. (2017). Efficient extraction of cellulose nanocrystals through hydrochloric acid hydrolysis catalyzed by inorganic chlorides under hydrothermal conditions. ACS Sustain. Chem. Eng..

[39] Mohammed M.A., Basirun W.J., Rahman N.M.M.A., Salleh N.M. (2021). The effect of particle size of almond shell powders, temperature and time on the extraction of cellulose. J. Nat. Fibers..

[40] Agustin M.B., Ahmmad B., De Leon E.R.P., Buenaobra J.L., Salazar J.R., Hirose F. (2013). Starch-based biocomposite films reinforced with cellulose nanocrystals from garlic stalks. Polym. Compos..

[41] Elazzouzi-Hafraoui S., Nishiyama Y., Putaux J.L., Heux L., Dubreuil F., Rochas C. (2008). The shape and size distribution of crystalline nanoparticles prepared by acid hydrolysis of native cellulose. Biomacromolecules.

[42] Kaushik M., Chen W.C., van de Ven T.G., Moores A. (2014). An improved methodology for imaging cellulose nanocrystals by transmission electron microscopy. Nord. Pulp. Pap. Res. J..

[43] Coelho C.C., Michelin M., Cerqueira M.A., Gonçalves C., Tonon R.V., Pastrana L.M., Teixeira J.A. (2018). Cellulose nanocrystals from grape pomace: Production, properties and cytotoxicity assessment. Carbohydr. Polym..

[44] Neto W.P.F., Silvério H.A., Dantas N.O., Pasquini D. (2013). Extraction and characterization of cellulose nanocrystals from agro-industrial residue–Soy hulls. Ind. Crops Prod..

[45] Roman M., Winter W.T. (2004). Effect of sulfate groups from sulfuric acid hydrolysis on the thermal degradation behavior of bacterial cellulose. Biomacromolecules.

[46] Martínez-Sanz E., Ossipov D.A., Hilborn J., Larsson S., Jonsson K.B., Varghese O.P. (2011). Bone reservoir: Injectable hyaluronic acid hydrogel for minimal invasive bone augmentation. J. Control Release.

[47] Morán J.I., Alvarez V.A., Cyras V.P., Vázquez A. (2008). Extraction of cellulose and preparation of nanocellulose from sisal fibers. Cellulose.

[48] de Morais Teixeira E., Bondancia T.J., Teodoro K.B.R., Corrêa A.C., Marconcini J.M., Mattoso L.H.C. (2011). Sugarcane bagasse whiskers: Extraction and characterizations. Ind. Crops. Prod..

[49] Cherian B.M., Leão A.L., de Souza S.F., Thomas S., Pothan L.A., Kottaisamy M. (2010). Isolation of nanocellulose from pineapple leaf fibres by steam explosion. Carbohydr. Polym..

[50] Sun B., Zhang M., Hou Q., Liu R., Wu T., Si C. (2016). Further characterization of cellulose nanocrystal (CNC) preparation from sulfuric acid hydrolysis of cotton fibers. Cellulose.

